# Assembly and comparative analysis of the first complete mitochondrial genome of *Lophophora williamsii* (Cactaceae)

**DOI:** 10.3389/fpls.2026.1870314

**Published:** 2026-06-02

**Authors:** Xingliang Liu, Ying Zhang, Yikai Wang, Shan Gao, Nan Li, Zhe Cui, Lifeng Zhang, Teng Zhang

**Affiliations:** 1 Beijing Key Laboratory of Psychoactive Substances Detection and Control, Beijing Narcotics Control Technology Center, Beijing, China; 2 National Anti-Drug Laboratory Beijing Regional Center, Beijing, China; 3 Ministry of Public Security Institute of Forensic Science, Beijing, China; 4 Science and Technology Research Center of China Customs, Beijing, China

**Keywords:** *Lophophora williamsii*, mitochondrial genome, genome architecture, RNA editing, intracellular gene transfer, phylogeny

## Abstract

**Introduction:**

The Cactaceae family exhibits remarkable adaptation to arid environments, yet mitochondrial genomic resources remain extremely scarce for this lineage. *Lophophora williamsii* (peyote) is an ecologically and culturally significant cactus species native to the Chihuahuan Desert, but its mitochondrial genome has never been characterized.

**Methods:**

We assembled and annotated the first complete mitochondrial genome of *L. williamsii* using PacBio HiFi long-read sequencing, and conducted comprehensive analyses including repeat identification, codon usage, RNA editing prediction, intracellular gene transfer detection, phylogenetic reconstruction, selection pressure assessment, and synteny comparison.

**Results:**

The assembled mitogenome is exceptionally large (2,422,778 bp) and structurally complex, comprising 57 contigs that resolve into five linear molecules. It encodes 33 core and 13 variable protein-coding genes, three rRNA genes, and 49 tRNA genes. A total of 587 simple sequence repeats (SSRs), 124 tandem repeats, and 5,519 interspersed repeats (≥30 bp) were identified. Codon usage bias is predominantly driven by natural selection. We predicted 431 C-to-U RNA editing sites across 32 PCGs and detected 18 chloroplast-derived DNA fragments (31,614 bp) containing 19 intact genes. Phylogenetic analysis based on 30 conserved mitochondrial PCGs placed *L. williamsii* as sister to *Mammillaria huitzilopochtli*. Most mitochondrial genes are under purifying selection, whereas *atp8* and *matR* exhibit positive selection. Synteny analysis revealed extensive collinear blocks decreasing with phylogenetic distance.

**Discussion:**

This first complete mitogenome of *L. williamsii* provides a fundamental genomic resource for understanding mitochondrial evolution in Cactaceae, offers high-resolution molecular markers for species identification and forensic authentication, and supports conservation genetics for this threatened species.

## 1 Introduction

Mitochondria are semi-autonomous organelles derived from ancient endosymbiotic α-proteobacteria and constitute the primary site of aerobic respiration and ATP synthesis in eukaryotic cells (Manoj and Bazhin, 2021; Kwasniak-Owczarek and Janska, 2024; Speth et al., 2024). Beyond their central role in energy metabolism, plant mitochondria participate in numerous essential biological processes, including metabolite biosynthesis, programmed cell death, cytoplasmic male sterility (CMS), and abiotic/biotic stress responses (Kunji et al., 2016; Ruprecht et al., 2019; Yang et al., 2022). Plant mitochondrial genomes (mitogenomes) are distinguished by their remarkable structural and evolutionary complexity. They vary drastically in size from approximately 66 kb in the parasitic plant *Viscum scurruloideum* to 11.7 Mb in *Larix sibirica*, undergo frequent structural rearrangements, contain abundant repetitive sequences, and frequently incorporate foreign DNA via intracellular gene transfer (IGT) and horizontal gene transfer (HGT) (Wang et al., 2023; Zhou et al., 2023b; Zhou et al., 2023; Lu and Li, 2024). These unique features make mitogenomes valuable resources for studies of plant phylogeny, evolution, genetic diversity, and cytoplasmic inheritance (Liu et al., 2016; Chen et al., 2017; Bi et al., 2020; Zhang et al., 2024).

Advances in next-generation sequencing (NGS) and long-read sequencing technologies have substantially accelerated the decoding of plant mitogenomes (Jin et al., 2020; Chen et al., 2025; Jain et al., 2025; Wang et al., 2025). Nevertheless, compared with chloroplast and nuclear genomes, mitochondrial genomes remain underrepresented due to the inherent assembly difficulties caused by frequent recombination, large repetitive regions, and multi-chromosomal structures (Szandar et al., 2022; Zhou et al., 2023a; Qu et al., 2024).

Cactaceae, one of the most species-rich angiosperm families in arid ecosystems, exhibits remarkable adaptations to water-limited environments (Bhau and Wakhlu, 2015; Carpena et al., 2023). Within this family, the genus *Lophophora* comprises two formally recognized species, *L. williamsii* and *L. diffusa*, which differ markedly in morphology, phytochemistry, and genetic composition (Ibarra-Laclette et al., 2015; Adrienne et al., 2016; Hwang et al., 2025). *Lophophora williamsii* (Lem. Ex. Salm-Dyck) J.M. Coulter, commonly known as peyote, is a spineless, globular cactus endemic to the Chihuahuan Desert and Tamaulipan thornscrub regions of northern Mexico and southern Texas, USA (Ibarra-Laclette et al., 2015; Soto-Trejo et al., 2024). As a culturally iconic plant with profound historical, religious, and medicinal significance to North American indigenous communities, *L. williamsii* has long been a focus of scientific and ethnobotanical interest, yet it faces mounting conservation pressure due to illegal harvesting and habitat fragmentation (Yesson et al., 2011). It typically displays blue-green stems with prominent ribs, pink flowers, and a high content of the psychoactive alkaloid mescaline, whereas *L. diffusa* has yellow-green stems without obvious ribs, white flowers, and negligible mescaline content. These phytochemical and morphological distinctions underpin the species’ contrasting legal regulatory statuses and economic values (Ibarra-Laclette et al., 2015; Adrienne et al., 2016; Hwang et al., 2025).

To date, genomic research on the *Lophophora* genus has focused primarily on chloroplast genomes. Cactus plastomes are known for extensive structural reduction, including the loss of inverted repeat regions and the entire *ndh* gene suite, features that are considered an adaptive signature to arid environments (Daniell et al., 2016; Kusnetsov, 2018; Cauz-Santos et al., 2020; Yu et al., 2023). Previous genomic studies on *L. williamsii* have been limited to nuclear transcriptome sequencing and were focused on the biosynthetic pathway of mescaline (Ibarra-Laclette et al., 2015), leaving its mitochondrial genome entirely unexplored. Chloroplast intergenic fragments such as *trnL/trnF* and *rbcL* have been widely adopted as core markers for species identification and forensic authentication of *Lophophora* specimens, owing to their moderate evolutionary rates (Adrienne et al., 2016; Hwang et al., 2025).

In stark contrast to the well-characterized chloroplast genomes, mitochondrial genomics within the Cactaceae family remains severely understudied. Although plant mitochondrial genomes are increasingly recognized as highly dynamic and structurally diverse, current molecular identification methods for *Lophophora congeners* still rely almost exclusively on short chloroplast gene fragments or nuclear simple sequence repeats. These markers frequently lack sufficient discriminatory power toward closely related taxa, and no organelle-level genomic evidence has yet been established to support robust and accurate species delimitation (Yesson et al., 2011).

Here, we present the first complete mitochondrial genome of *L. williamsii*, which fills the critical gap in mitochondrial genomic resources for the *Lophophora* genus. This mitogenome provides new opportunities to investigate mitochondrial genome architecture, gene evolution, and cytoplasmic inheritance patterns in this culturally and ecologically important cactus species. Moreover, it offers high-resolution molecular markers to facilitate accurate species identification and forensic authentication. This study lays a fundamental genomic foundation for the conservation genetics and sustainable management of this vulnerable cactus species, which is under increasing threat from illegal collection and habitat degradation.

## 2 Materials and methods

### 2.1 Plant materials sampling, DNA extraction, and sequencing

Fresh stem epidermal tissues of *Lophophora williamsii* were collected from a specimen intercepted by China Customs and subsequently maintained under controlled conditions in the laboratory of the Science and Technology Research Center of China Customs in Beijing, China (39.9°N, 116.3°E). As *L. williamsii* is not native to China, the sampled plant originated from an intercepted import and was cultivated under laboratory conditions exclusively for research purposes. Voucher specimens were deposited in the Science and Technology Research Center of China Customs (voucher number: WYY20240510-02; contact person: Yikai Wang; email: wangyikai_2001@163.com). Epidermal tissue from the stem was collected from *Lophophora williamsii* plants. Immediately following sampling, the samples were flash-frozen in liquid nitrogen and then stored at -80°C until processing. Total genomic DNA was isolated using an optimized cetyltrimethylammonium bromide (CTAB)-based protocol. DNA concentration and purity were assessed using a NanoDrop 1000 spectrophotometer and a Qubit fluorometer (Thermo Fisher Scientific). A 15-kb SMRTbell library was constructed using a SMRTbell Express Template Prep Kit 2.0 (Pacific Biosciences, CA, USA), including DNA shearing, AMPure PB bead purification, ssDNA overhang removal, damage repair, end repair, hairpin adapter ligation, and library bead purification. Following quality control, the library was sequenced on the PacBio Revio platform (Pacific Biosciences, CA, USA) by Shenzhen Huitong Biotechnology Co., Ltd. Raw reads were processed using the CCS algorithm (version 6.0.0, parameters: ‐‐min Passes 3 ‐ min Predicted Accuracy 0.99 ‐‐max Length 21,000) to generate highly accurate HiFi reads.

### 2.2 Assembly and annotation of the mitochondrial genome

Mitochondrial genome assembly was performed using PacBio HiFi reads (sequencing depth exceeding 5 Gb; average read length 12.32 kb) with the PMAT software (v2.0.1). The preliminary assembly graph was visualized and manually adjusted using Bandage (v0.8.1). HiFi reads were then aligned to the draft assembly by minimap2, followed by further error correction and sequence refinement using NextPolish (v1.3.1; https://github.com/Nextomics/NextPolish) to generate the final high−accuracy assembly. The corresponding assembly graph file was adjusted accordingly. Mitochondrial genome annotation was performed using MITOFY (Alverson et al., 2010) and MFANNOT (Beck and Lang, 2010), and its map was generated with OGDRAW (Greiner et al., 2019).

### 2.3 Analysis of repeat sequences

Simple sequence repeats (SSRs) were identified using the MISA tool (v1.0; parameters: 1 10, 2 5, 3 4, 4 3, 5 3, 6 3, respectively) (Thiel et al., 2003). Tandem repeats were detected using the TRF software (TRF409. linux64; parameters: 2 7 7 80 10 50 2000 -f -d -m) (Benson, 1999). Interspersed repeats were analyzed using the REPuter software (Kurtz et al., 2001), via its online server (https://bibiserv.cebitec.uni-bielefeld.de/reputer/), with a minimum repeat size of 30 bp and a sequence identity of 90% (equivalent to a Hamming distance of 3). All repeat elements were visualized using Circos v0.69-8 (Zhang et al., 2013).

### 2.4 Analysis of relative synonymous codon usage

To ensure data integrity, redundant sequences and coding regions shorter than 300 bp were excluded from the analysis. Only protein-coding sequences starting with the initiation codon (ATG) and terminated by a canonical stop codon (TAA, TAG, or TGA) were retained. Codon usage indices, including GC content, relative synonymous codon usage (RSCU), and effective number of codons (ENC), were calculated using CodonWv1.4.4 (Sharp and Li, 1986).

To assess codon usage bias, an ENC-plot was generated by plotting the effective number of codons (ENC) against the GC content at the third synonymous codon position (GC3s). Theoretical ENC values were derived using Equation 1 to establish a standard reference curve (Romero et al., 2000). To further evaluate the deviation between observed and expected ENC values, the ENC ratio was calculated according to Equation 2, and the frequency distribution of these ratios was subsequently analyzed. The formulas used were as follows:

(1) $$\text{ENC}=\;2+GC3\;+\;29/[GC3^{2}+{\left(1-GC3\right)}^{2}]$$

(2) ENC ratio= (theoretical ENC - actual ENC)/actual ENC

Data points situated on or near the standard curve indicate mutational pressure as the primary driver of codon usage, whereas points falling below the curve suggest a stronger influence of natural selection (Sueoka, 1988). Additionally, a GC plot was constructed using the ‘ggplot2’ package, with the average GC content at the first and second codon positions (GC12) plotted on the y-axis against the GC content at the third position (GC3) on the x-axis. The diagonal line (y = x) was superimposed as a reference to facilitate subsequent analysis.

### 2.5 Identification of RNA editing sites and chloroplast-to-mitochondrial fragments

Chloroplast−to−mitochondrial intracellular gene transfer (IGT) events in *L. williamsii* were identified using BLASTN (v2.9.0+) (Altschul et al., 1990), with an e-value threshold of 1e-5 and a word size of 7. To ensure biological relevance, only homologous fragments with an alignment length greater than 1,000 bp were retained for further analysis. Genomic synteny relationships were visualized using Circos (v0.69-8) (Zhang et al., 2013). RNA editing sites in the PCGs were predicted using the PmtREP program with a cutoff value of 0.2 (Mower, 2005).

### 2.6 Analysis of phylogenetic

To determine the phylogenetic position of *L. williamsii*, mitochondrial genome sequences from 27 related species were retrieved from NCBI, with *Daucus carota subsp. Sativus*, *Batis maritima*, and *Helianthus occidentalis* designated as outgroups (**Supplementary Table 1**). Single-copy orthologous genes were extracted using OrthoFinder v2.5.5 with default parameters. OrthoFinder employs reciprocal best hits (RBH) for initial ortholog detection and performs phylogenetic clustering to identify high-confidence single-copy orthologs. Synteny information was not incorporated in this orthology inference pipeline. A total of 30 single-copy orthologous genes(*atp9*, *nad6*, *cox2*, *matR*, *nad1*, *nad9*, *nad5*, *nad2*, *cob*, *rpl5*, *nad4L*, *ccmB*, *ccmFc*, *atp1*, *rps12*, *cox1*, *nad7*, *nad3*, *atp8*, *mttB*, *atp6*, *atp4*, *cox3*, *ccmC*, *nad4*, *ccmFn*, *rps4*, *rps3*, *rps13*, and *rps7*) were retained for subsequent analyses. These sequences were aligned with MAFFT (v7.313) (Katoh and Standley, 2013). The resulting alignments were processed in Gblocks (0.91b) (Talavera and Castresana, 2007), applying default parameters with the exception of gap handling. Maximum likelihood phylogenetic reconstruction was performed using IQ-Tree (v3.0.1) (Nguyen et al., 2015). Model selection based on the Bayesian Information Criterion identified GTR+F+R3 as the optimal nucleotide substitution model with a bootstrap of 1000.

### 2.7 Analysis of Ka/Ks and nucleotide variability

To assess nonsynonymous (Ka) and synonymous (Ks) substitution rates, homologous gene pairs were identified between *Lophophora williamsii* (PZ264132, PZ264133, PZ264134, PZ264135, PZ264136) and four related species: *Mammillaria huitzilopochtli* (OP081771), *Selenicereus monacanthus* (OQ835513), *Opuntia cochenillifera* (OR885585), and *Pereskia aculeata* (ON496936). OrthoFinder v2.5.5 was used to identify shared single-copy orthologous core genes across the five species, rather than for raw sequence extraction. All putative orthologs were further validated to ensure the presence of intact open reading frames (ORFs) and full length coding sequences to exclude fragmented or incomplete genes caused by repetitive sequences in plant mitochondrial genomes. Finally, 28 complete homologous core genes were retained for Ka/Ks calculation (*atp1, atp4, atp6, atp8, atp9, ccmB, ccmC, ccmFc, ccmFn, cob, cox1, cox2, cox3, matR, mttB, nad1, nad2, nad3, nad4, nad4L, nad5, nad6, nad7, nad9, rpl5, rps12, rps13, rps4*). Pairwise comparisons were performed by aligning protein sequences of each target species to those of the reference species using Blastn (v2.9.0+) with an *E*-value threshold of 1e-5 and -max_target_seqs set to 1. Only reciprocal best hits were retained. The optimally matched pairs were subsequently extracted, and the protein and nucleotide sequences of the target and reference species were concatenated. Aligned sequences were formatted using ParaAT (v2.0) (Zhang et al., 2012) with default parameters. Finally, the KaKs_Calculator (v2.0) (Zhang et al., 2006) was employed to estimate the non-synonymous (Ka) and synonymous (Ks) substitution rates, as well as their corresponding Ka/Ks ratios, using the MLWL method. To comprehensively evaluate nucleotide variability across mitochondrial coding regions, homologous gene sequences from the five species were aligned using MAFFT (v 7.429) (Katoh and Standley, 2013). Finally, DnaSP (v 6.0) software was used for calculating the Nucleotide variability across the cp genome through a sliding window approach with a step size of 100 and a window length of 200 base pairs (Rozas et al., 2017).

### 2.8 Analysis of sequence collinearity

Homologous sequences between *L. williamsii* and the other 4 selected species (*M. huitzilopochtli*, *S. monacanthus*, *O. cochenillifera*, and *Pereskia aculeata*) were identified using BLASTN (v2.9.0+) (word size=7, E-value threshold=1e-5). Collinearity relationships were visualized using TBtools (v2.450).

## 3 Results

### 3.1 Assembly, and annotation of mitochondrial genome of *L. williamsii*

**Figure 1:**
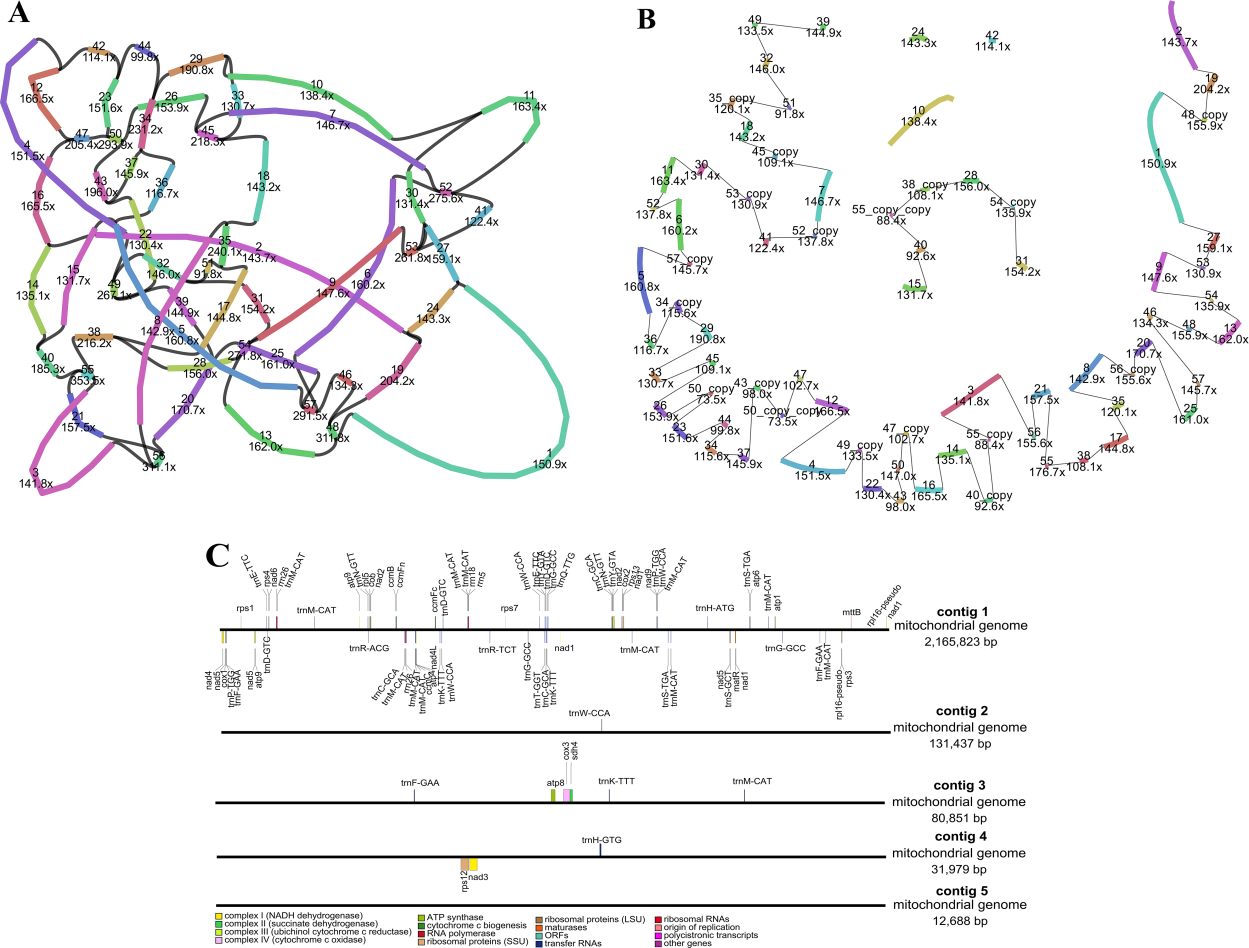
The structural features of *L. williamsii*. **(A)** Branched conformation of *L. williamsii* mitochondrial genome. **(B)** Contiguous sequences of *L. williamsii* mitochondrial genome after de-catenation. **(C)** The mitochondrial genome maps of the *L. williamsii* contig1 to contig5.

The complete mitochondrial genome of *L. williamsii* exhibited a multi-branched conformation (**Figure 1A**) with a total length of 2,422,778 bp and a GC content of 42.84%. The raw assembly graph presented a multi−branched closed conformation consisting of 57 nodes (**Figure 1B**). The graph was visualized using Bandage based on PacBio HiFi sequencing data, with each node labeled by node number and sequencing coverage depth. Among these, nodes 34, 35, 38, 40, 43, 45, 47, 49, 50, 52, 53, 54, 55, 56, and 57 were predicted repeat regions potentially involved in mediating mitochondrial genome recombination. For subsequent analysis, the multi−branched closed graph was decatenated into five linear mitochondrial molecules, assembled according to the following node sequences:

Contig1: 2→19→48_copy→1→27→53→9→54→13→48→46→57→25→20→56_copy→8→35→17→38→55→21→56→3→55_copy→40_copy→14→16→47_copy→50→43→22→49_copy→4→12→47→50_copy_copy→43_copy→37→34→44→23→50_copy→26→45→33→29→34_copy→36→5→57_copy→6→52→11→30→53_copy→41→52_copy→7→45_copy→18→35_copy→51→32→49→39.

Contig2: 15→40→55_copy_copy→38_copy→28→54_copy→31.

Contig3: 10.

Contig4: 24.

Contig5: 42.

The final assembled mitochondrial genome consisted of five linear chromosomes (hereafter referred to as chromosomes 1–5) with a combined length of 2,422,778 bp. Chromosomes 1–5 had lengths of 2165823 bp, 131437 bp, 80851 bp, 31979 bp, and 12688 bp, respectively, with GC contents of 42.87%, 42.36%, 43.18%, 42.08%, and 41.61%. *L. williamsii* mitochondrial genome sequences were deposited in the GenBank database under accession numbers PZ264132, PZ264133, PZ264134, PZ264135, and PZ264136.

Coverage analysis of the mitochondrial genome (**Supplementary Figure 1**) was performed by aligning the long-read sequencing data to the assembled mitogenome using BWA software (Li, 2013). Per−base sequencing depths were calculated using samtools depth (Li et al., 2009), and a coverage map was generated using a custom Python script, with genomic position plotted on the x−axis and sequencing depth on the y−axis. The results showed that the mitogenome was fully covered without gaps, with an average sequencing depth of 65.2×, confirming the accuracy and reliability of the assembly.

The mitochondrial genome of *L. williamsii* encoded 33 core PCGs, 13 variable PCGs, 3 rRNA genes, and 19 tRNA genes (**Figure 1C**). The PCG complement included nine NADH dehydrogenase genes (*nad1, nad2, nad3, nad4, nad4L, nad5, nad6, nad7*, *nad9*), five small subunit ribosomal protein genes (*rps1*, *rps3*, *rps4*, *rps7*, *rps12*, *rps13*) and two large subunit ribosomal protein genes (*rpl16*, *rpl5*), five ATP synthase genes (*atp1, atp4, atp6, atp8,atp9*), four cytochrome c biogenesis genes (*ccmB, ccmC, ccmFc, ccmFn*), three cytochrome c oxidase genes (*cox1, cox2,cox3*), two ribosomal protein genes (*rpl16, rpl5*), one ubiquinol cytochrome c reductase gene (*cob*), one maturation enzyme gene (*matR*), one succinate dehydrogenase gene (*sdh4*) and one transport membrane protein gene (*mttB*), 49 tRNA genes (*trn*C-GCA(×3), *trn*D-GTC(×3), *trn*E-TTC(×2) *trn*F-GAA(×2), *trn*F-GAA, *trn*G-GCC(×3), *trn*H-ATG, *trn*H-GTG, *trn*K-TTT(×2), *trn*K-TTT, *trn*M-CAT(×12), *trn*M-CAT, *trn*N-GTT(×2), *trn*P-TGG(×2), *trn*Q-TTG, *trn*R-ACG, *trn*R-TCT, *trn*S-GCT, *trn*S-TGA(×2), *trn*Y-GTA(×2), *trn*W-CCA (Kunji et al., 2016), *trn*T-GGT), and four rRNA genes (*rrn*5, *rrn*26 (×2), *rrn*18) (**Figure 1C**; **Table 1**).

**Table 1:** Gene composition of the *L. williamsii* mitochondrial genome.

Group of genes	Gene name
ATP synthase	*atp1 atp4 atp6 atp8 atp9* (2)
Cytochrome c biogenesis	*ccmB ccmC ccmFc ccmFn*
Ubiquinol cytochrome c reductase	*cob*
Cytochrome c oxidase	*cox1 cox2 cox3*
Maturases	*matR*
Transport membrane protein	*mttB*
NADH dehydrogenase	*nad1 nad2 nad3 nad4 nad4L nad5 nad6 nad7 nad9*
Ribosomal proteins (LSU)	*rpl16 rpl16 rpl5*
Ribosomal proteins (SSU)	*rps1 rps12 rps13 rps3 rps4 rps7*
Succinate dehydrogenase	*sdh4*
Ribosomal RNAs	*rrn18 rrn26* (2) *rrn5*
Transfer RNAs	*trnC-GCA* (3) *trnD-GTC* (3) *trnE-TTC* (2) *trnF-GAA* (2) trnF-G*AA trnG-GCC (3) trnH-ATG trnH-GTG trnK-TTT* (2) *trnK-TTT* trnM-CAT (12) trnM-CAT *trnN-GTT* (2) *trnP-TGG* (2) *trnQ-TTG trnR-ACG trnR-TCT trnS-GCT trnS-TGA* (2) *trnT-GGT trnW-CCA* (4) *trnY-GTA* (2)

Gene (2), Number of copies of multi-copy genes.

### 3.2 Repeat sequence analysis

**Figure 2:**
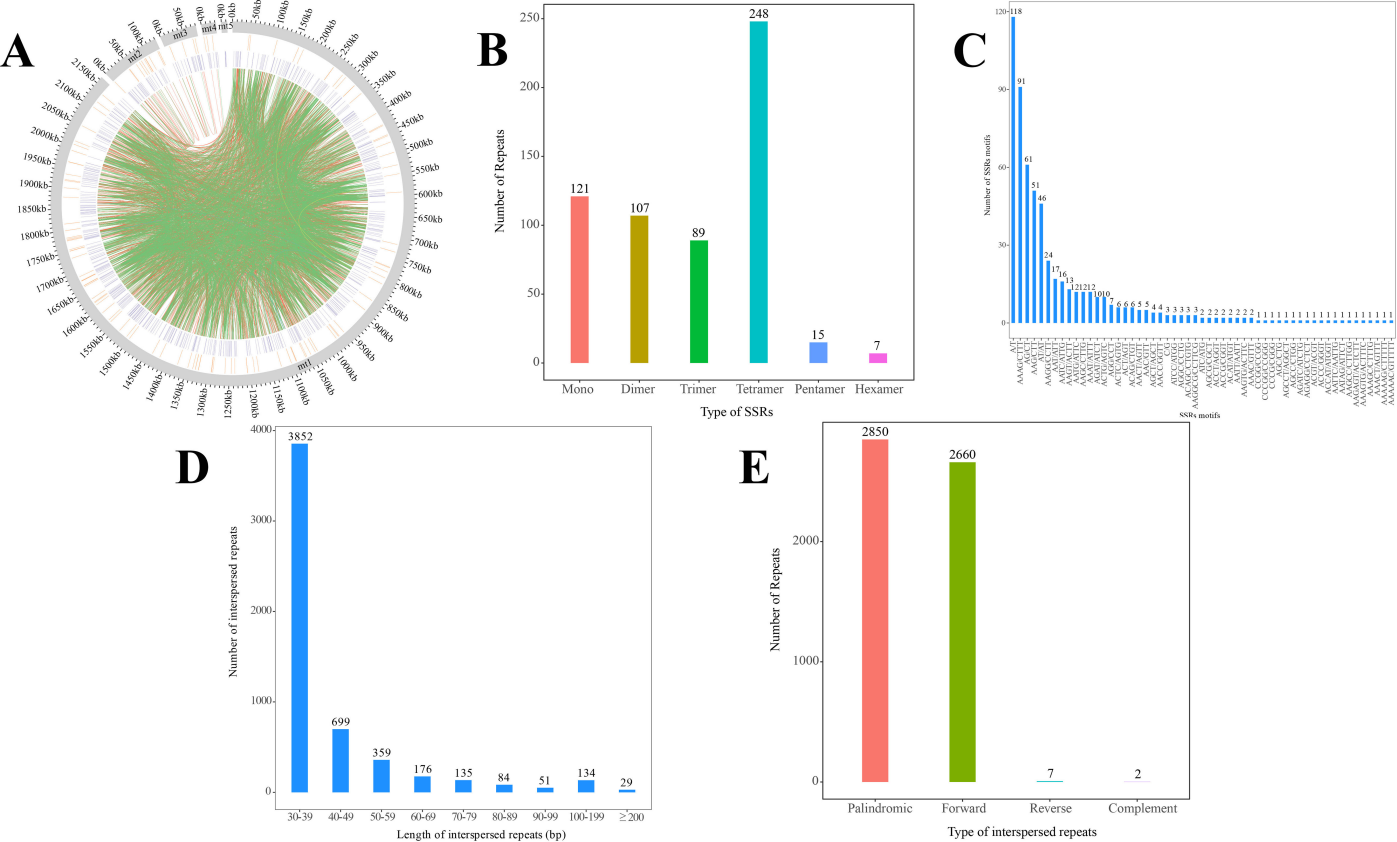
The repeats of *L. williamsii* mitochondrial genome. **(A)** The repeat sequences on the genome map. **(B)** Type and number of SSRs repeats. **(C)** Type and number of SSRs motifs. **(D)** The length distribution of interspersed repeats. **(E)** Type and number of interspersed repeats.

A total of 587 simple sequence repeats (SSRs) were detected in the mitochondrial genome of *L. williamsii* (**Figure 2A**). These comprised 121 mononucleotide (20.61%), 107 dinucleotide (18.23%), 89 trinucleotide (15.16%), 248 tetranucleotide (42.25%), 15 pentanucleotide (2.56%), and 7 hexanucleotide (1.19%) repeats (**Figure 2B**). A/T mononucleotide repeats were the most abundant type of SSRs, with a total of 118 loci, accounting for 97.52% of all mononucleotide SSRs. They were followed by AAAG/CTTT tetranucleotide repeats, with 91 loci in total (**Figure 2C**). A total of 124 tandem repeats were identified, with sequence identities ≥ 61% and lengths ranging from 4–39 bp (**Figure 2D**). Interspersed repeat analysis revealed 5519 pairs of repeats ≥ 30 bp in length, including 2660 pairs of forward repeats, 2850 pairs of palindromic repeats, 7 pairs of reverse repeats, and 2 pairs of complement repeats (**Figure 2E**).

### 3.3 Codon usage analysis

**Figure 3:**
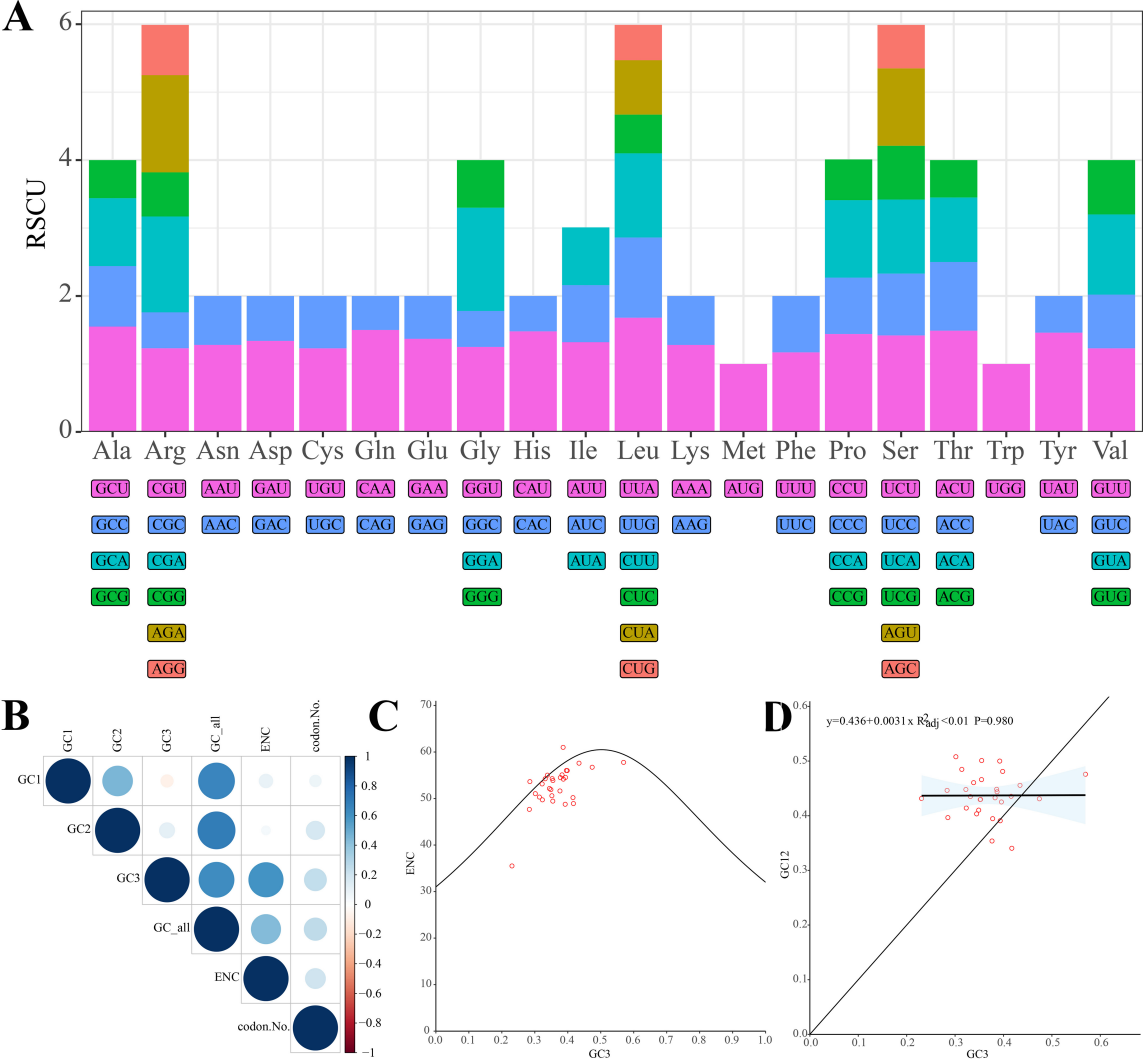
Analysis of relative synonymous codon usage. **(A)** Relative synonymous codon usage (RSCU) in *L. williamsii* mitochondrial genomes. The X-axis shows amino acids, and the Y-axis shows RSCU values for codons. **(B)** Correlation of GC content and ENC value. **(C)** ENC plot. **(D)** Neutrality plot.

To assess codon usage bias in the mitochondrial genome of *L. williamsii*, we analyzed 29 protein-coding genes (PCGs): *atp1, atp4, atp6, atp8, atp9, ccmB, ccmC, ccmFc, ccmFn, cob, cox1, cox2, cox3, matR, mttB, nad2, nad3, nad4, nad5, nad6, nad7, nad9, rpl5, rps1, rps12, rps13, rps3, rps4*, and *rps7* (**Figure 3A**). Codons with relative synonymous codon usage (RSCU) values > 1 were considered preferentially used for their corresponding amino acid. Widespread codon usage bias was observed across *L. williamsii* mitochondrial PCGs, with the only exceptions being the start codon (AUG), tryptophan (UGG), and alanine (GCA), each with an RSCU value of 1. In total, 5359 codons exhibited RSCU values > 1, confirming their preferential usage in *L. williamsii* mitochondrial genes. These high-frequency codons (RSCU > 1) predominantly featured A or U at the third position, whereas low-frequency codons (RSCU < 1) primarily ended with G or C, consistent with codon usage trends in terrestrial plant organelles. Leucine (Leu) has the strongest bias toward UUA, which had the highest RSCU value (1.68) among all codons in mitochondrial PCGs.

CodonW analysis revealed distinct nucleotide composition biases across codon positions (**Figure 3B**). The average GC content at the first (GC1), second (GC2), and third (GC3) codon positions was 46.94% (range: 32.52-58.04%), 40.47% (range: 32.64-48.41%), and 36.68% (range: 23.08-56.93%), respectively. The overall GC content (GCall) averaged 41.36% (range: 35.95-50.70%). This hierarchical distribution (GC1 > GC2 > GC3) reflects preferential enrichment of C/G nucleotides at the second codon position.

Correlation analysis uncovered intricate interactions between nucleotide composition at different codon positions and codon usage bias in the *L. williamsii* mitochondrial genome. GC1 was significantly positively correlated with GC2 (*P* < 0.05) and GCall (*P* < 0.001), but negatively correlated with GC3. GC2 showed positive correlations with GC3 and a highly significant correlation with GCall (*P* < 0.001). Most notably, the effective number of codons (ENC) was highly significantly positively correlated with both GC3 and GCall (*P* < 0.001), whereas correlations with GC1 and GC2 were not statistically significant. These results indicate that codon usage bias in the *L. williamsii* mitochondrial genome is shaped predominantly by mutational pressure acting on the third codon position and by overall genomic GC content, with comparatively minor contributions from the first and second codon positions.

The ENC-GC3 plot revealed a wide dispersion of *L. williamsii* mitochondrial genes (**Figure 3C**). Most genes were positioned well below the theoretical expectation curve, indicating that their codon usage patterns are predominantly shaped by natural selection. In contrast, a smaller subset of genes clustered near or slightly above the expected curve, suggesting a stronger influence of mutational pressure on their codon bias. Quantitative analysis revealed that 45% of genes (n=13) exhibited ENC ratios between -0.05 and 0.05, followed by 12 genes (the second largest group) in the 0.05–0.15 interval. The considerable deviation of observed ENC values from expected values for most genes suggests that codon usage bias in the *L. williamsii* mitochondrial genome is governed by weak mutational pressure but strong natural selection. Taken together, both mutational pressure and natural selection contribute to shaping codon usage patterns in the *L. williamsii* mitochondrial genome, with natural selection acting as the dominant evolutionary force.

Neutrality plot analysis (**Figure 3D**) demonstrated broad variation in GC content at the first and second codon positions (GC12: 0.340–0.508) and at the third position (GC3: 0.231-0.569). Linear regression analysis yielded a slope of 0.0031, indicating a negligible correlation between GC3 and GC12. This near-zero slope suggests that mutational pressure affects base composition at the third codon position differently from that at the first and second positions. Thus, mutational bias plays only a minor role in shaping codon usage patterns in the *L. williamsii* mitochondrial genome.

### 3.4 Prediction of RNA editing sites and DNA migration from chloroplast to mitochondria

**Figure 4:**
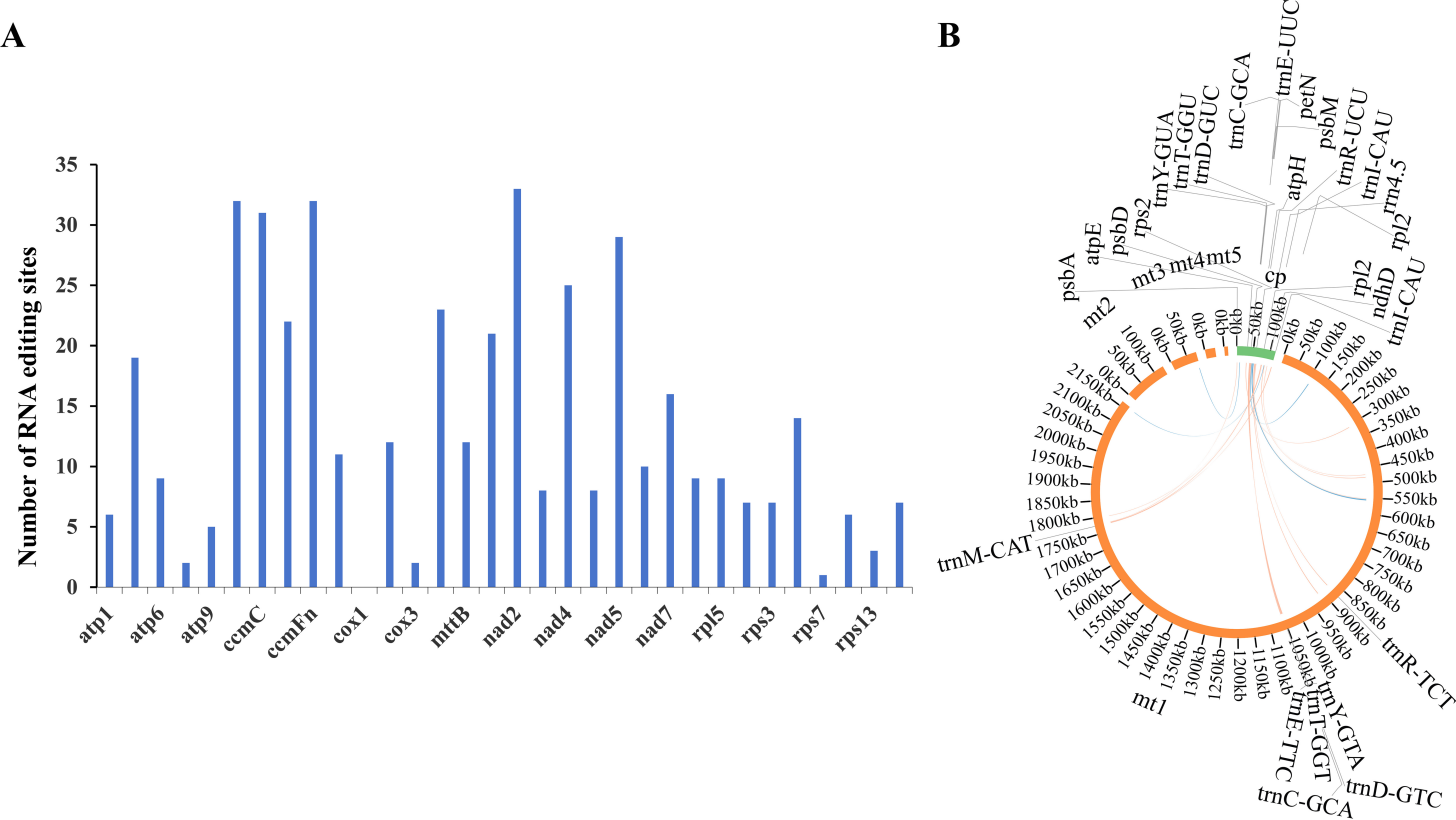
**(A)** Number of predicted RNA editing sites in PCGs. **(B)** Sequence migration analysis (the orange arc represents the mitochondrial genome, and the green arc represents the chloroplast genome. The blue and pink arcs inside the circle indicate homologous regions between the mitochondrial and chloroplast genomes. Genes labeled in the figure are complete genes identified on long homologous fragments.).

Transcriptome-wide prediction identified 431 RNA editing sites distributed across 32 PCGs in the *L. williamsii* mitochondrial genome (**Figure 4A**). All predicted events were C-to-U conversions and encompassed key metabolic genes, including members of the *atp* and *ccm* families, as well as *nad* and *cox* subunits. Editing site distribution exhibited marked heterogeneity: the *nad2* gene exhibited the greatest number of editing events (33 sites), whereas *cox1* showed no detectable editing. Notably, *rps7* contained a solitary editing site, representing the lowest frequency among the edited genes.

Sequence similarity analysis identified 18 mitochondrial DNA fragments putatively derived from the chloroplast genome (**Figure 4B**). These long fragments exceeded 1,000 bp in alignment length, totaling 31 614 bp, with the longest homologous fragment measuring 3670 bp. Annotation of these homologous sequences revealed 19 complete chloroplast genes distributed across the 18 long homologous fragments, including 10 PCGs (*atpE, atpH, ndhD, petN, psbA, psbD, psbM, rpl2* (×2), *rps2*), 8 tRNA genes (*trnC*-GCA, *trnD*-GUC, *trnE*-UUC, *trnI*-CAU (×2), *trnR*-UCU, *trnT*-GGU, *trnY*-GUA), and 1 rRNA gene (*rrn4.5*).

### 3.5 Phylogenetic analysis

**Figure 5:**
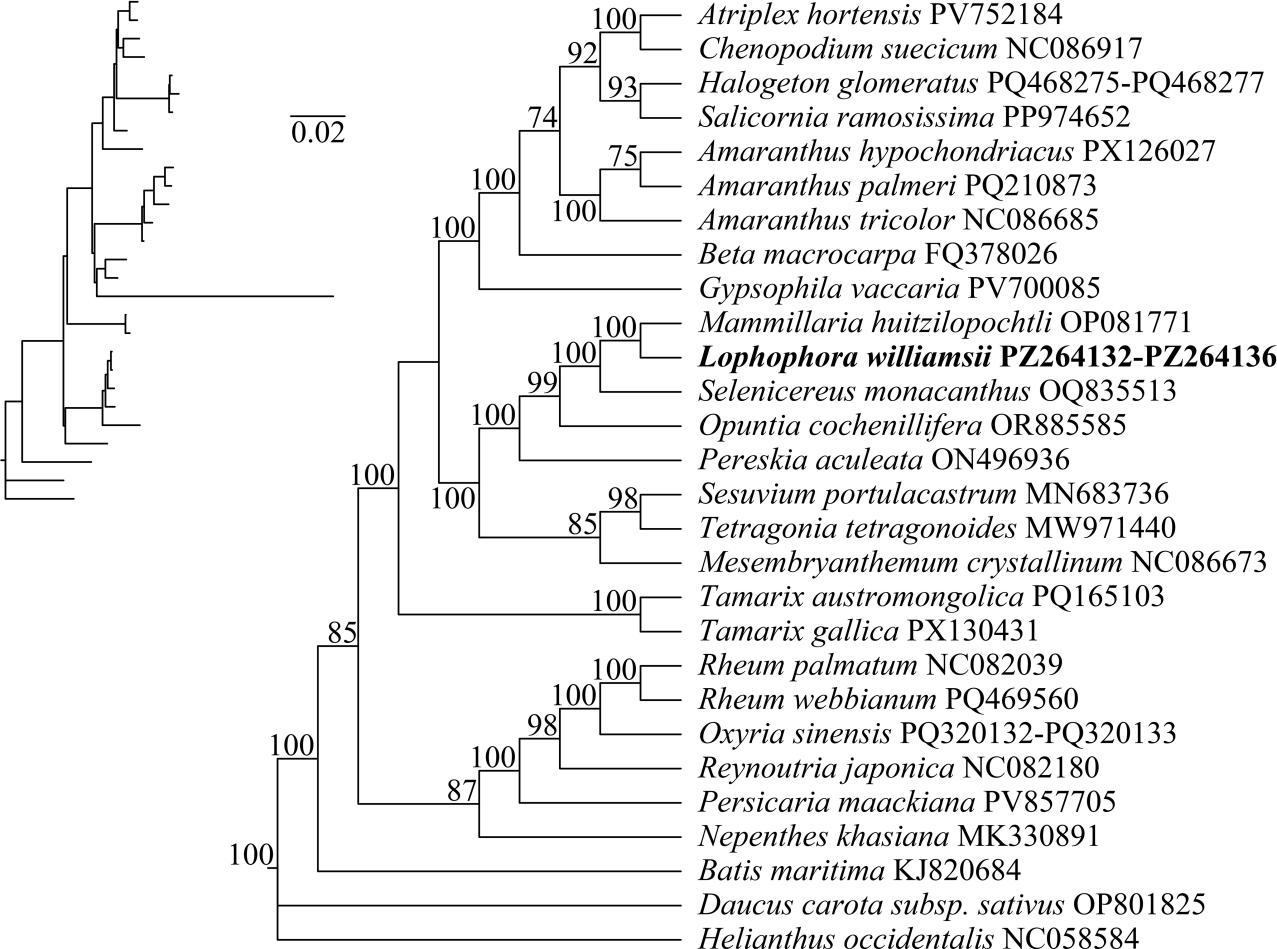
The phylogenetic relationships of *L. williamsii* with other 27 plant species by maximum likelihood based on the single-copy orthologous genes from mitochondrial genomes.

Phylogenetic reconstruction based on concatenated DNA sequences from 30 mitochondrial PCGs across 28 species yielded a well-resolved topology, with the vast majority of branches supported by bootstrap values ≥ 85% and most achieving maximum statistical confidence (100%). Within this framework, the five representative Cactaceae species resolved into a strongly supported monophyletic clade (99-100%), within which *L. williamsii* was identified as the sister lineage to *Mammillaria huitzilopochtli*. Furthermore, the Cactaceae clade was positioned as sister to Aizoaceae (**Figure 5**). This inferred topology aligns precisely with the established ordinal classification of the Caryophyllales, reinforcing the congruence between mitochondrial genomic data and current angiosperm systematics.

### 3.6 Ka/Ks and nucleotide variability analysis

**Figure 6:**
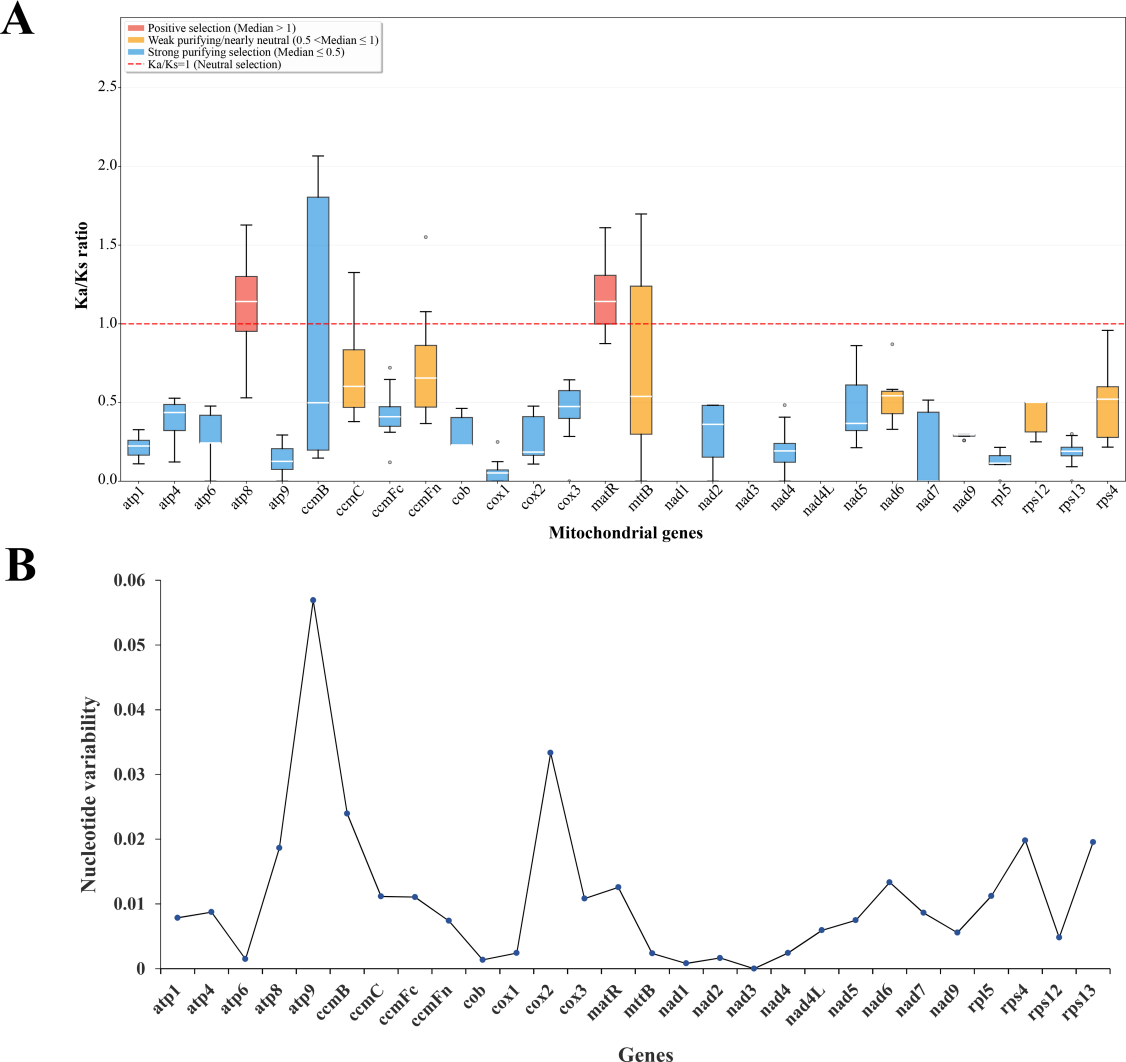
**(A)** Analysis of the Ka/Ks ratios for 28PCGs (Red indicates positive selection (median > 1), orange indicates weak purifying/near-neutral selection (0.5 < median ≤ 1), and blue indicates strong purifying selection (median ≤ 0.5), the red dashed line denotes Ka/Ks = 1). **(B)** Nucleotide variability of *L. williamsii* mitochondrial genome.

Ka/Ks analysis across 28 homologous core mitochondrial genes in five species yielded 280 pairwise estimates, with 47 missing data points (**Figure 6A**). To ensure statistical rigor, genes with fewer than five valid comparisons (e.g., nad3, containing 10 NA values) were excluded, retaining 27 reliable genes for downstream analysis: *atp*1, *atp*4, *atp*6, *atp*8, *atp*9, *ccm*B, *ccm*C, *ccm*Fc, *ccm*Fn, *cob*, *cox*1, *cox*2, *cox*3, *mat*R, *mtt*B, *nad*1, *nad*2, *nad*4, *nad*4L, *nad*5, *nad*6, *nad*7, *nad*9, *rpl*5, *rps*12, *rps*13, *rps*4. The majority of mitochondrial genes (19 in total, including *atp*1, *atp*4, *atp*6, *atp*9, *ccm*B, *ccm*Fc, *cob*, *cox*1, *cox*2, *cox*3, *nad*1, *nad*2, *nad*3, *nad*4, *nad*4L, *nad*5, *nad*7, *rp*l5, *rps*13) exhibited median Ka/Ks ratios ≤ 0.5, indicative of strong purifying selection and high functional conservation. Six genes (*ccm*C, *ccm*Fn, *mtt*B, *nad*6, *rps*12, *rps*4) showed median ratios between 0.5 and 1, consistent with moderately relaxed selective constraints. In contrast, *atp*8 and *mat*R displayed median Ka/Ks ratios >1, with box plots consistently positioned above the neutral threshold (Ka/Ks = 1). These results suggest that *atp*8 and *mat*R have undergone significant positive selection, implicating them as potential drivers of environmental adaptation or species divergence.

Nucleotide variability analysis across the same 28 core mitochondrial genes in five species identified *atp*9 as the most variable locus (Pi = 0.05689), followed by *cox*2 (Pi = 0.03333) and *ccm*B (Pi = 0.02395) (**Figure 6B**). Conversely, *nad3* was completely conserved (Pi = 0). With the singular exception of *atp*9, all 27 remaining PCGs displayed Pi values below the 0.05 threshold, underscoring the profound evolutionary constraint and overall sequence stability of the mitochondrial genome.

### 3.7 Analysis of sequence collinearity

**Figure 7:**
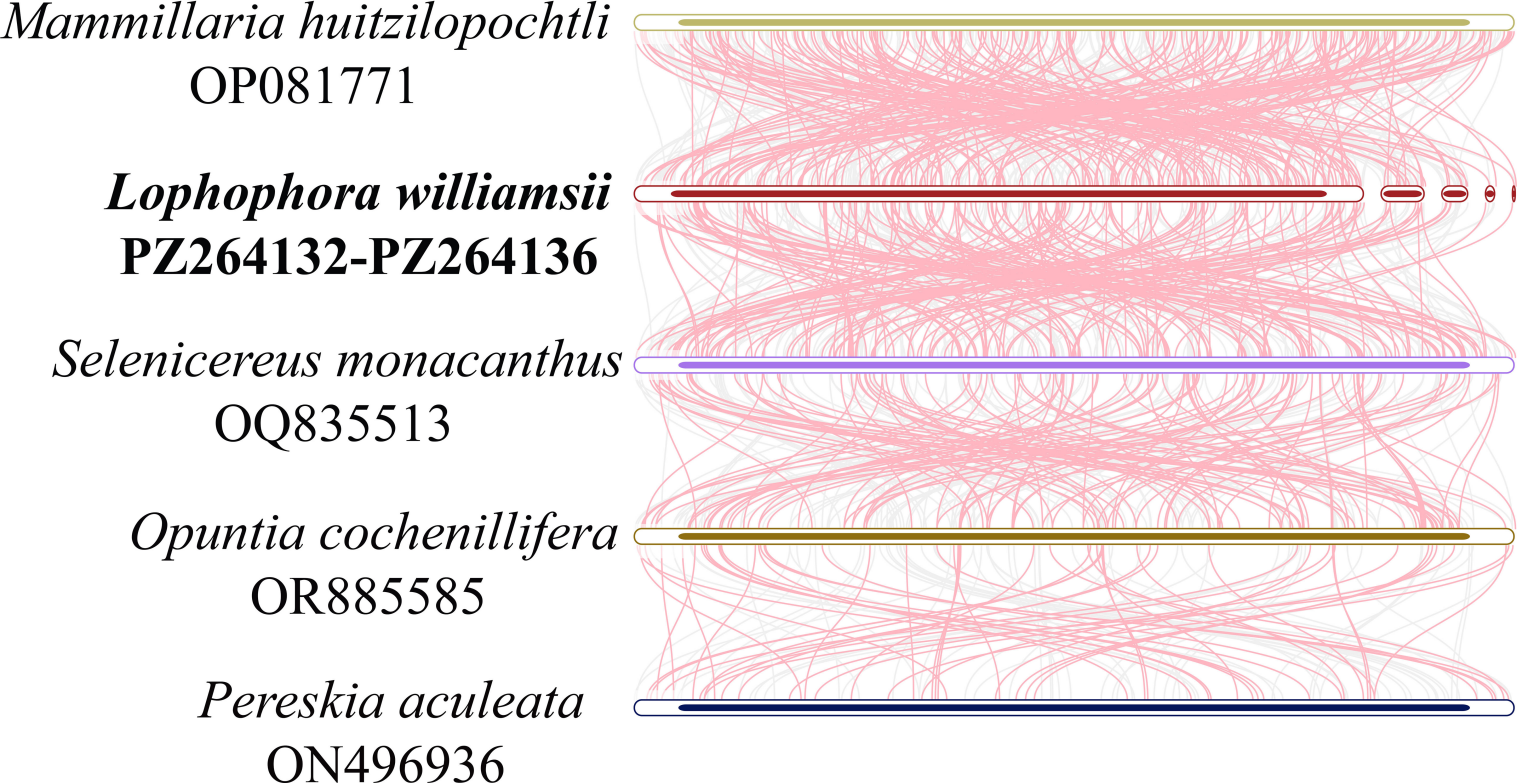
Analysis of sequence collinearity (the regions connected by arcs in the figure indicate areas with high homology, red arcs represent inverted sequences, while gray regions represent forward sequences).

Synteny analysis (**Figure 7**) identified extensive homologous segments across the five species, with red arcs representing inverted alignments and gray arcs representing forward-oriented regions. Genomic intervals lacking collinearity correspond to species-specific loci. Numerous collinear blocks exceeding 1,000 bp were detected. Specifically, pairwise comparisons revealed 495 (1,410,338 bp) collinear blocks between *M. huitzilopochtli* and *L. williamsi*, 401 (972,536 bp) between *L. williamsii* and *S. monacanthus*, 251 (561,110 bp) between *S. monacanthus* and *O. cochenillifera*, and 115 (353,433 bp) between *O. cochenillifera* and *P. aculeata*. These findings reveal extensive homologous collinear blocks shared among the Cactaceae species examined. Notably, a general trend of progressive decline in both block number and cumulative length was observed with increasing phylogenetic distance, consistent with the expectation that sequence homology erodes over evolutionary time.

### 3.8 Analysis of gene loss

**Figure 8:**
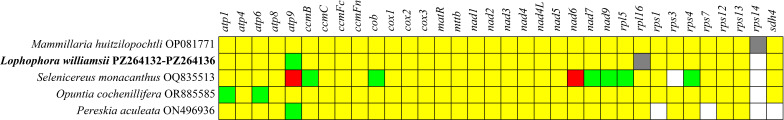
Analysis of gene loss (the PCGs distribution of *L. williamsii* and other four species. Yellow boxes indicated that one copies in the mitochondrial genomes. White boxes indicated that the gene was absent in the mitochondrial genomes. Grey boxes indicated that the gene was pseudogene. Green and red indicated that distinct gene copies).

Comparative analysis of PCG distribution across the five mitochondrial genomes revealed a mosaic of evolutionary conservation and lineage-specific variation (**Figure 8**). While core bioenergetic modules, including ATP synthase, cytochrome *c* biosynthesis and reductase, maturases, membrane transport proteins, and NADH dehydrogenase, remained largely conserved, genes encoding ribosomal proteins and succinate dehydrogenase exhibited substantial variability. Specifically, *rpl16*, *rps1*, *rps3*, *rps7*, *rps14*, and *sdh4* showed sporadic loss, whereas *rpl2*, *rpl10*, *rps2*, *rps10*, *rps11*, *rps19*, and *sdh3* were universally absent across all five species. This systematic depletion of ribosomal protein genes aligns with the established evolutionary trajectory of plant mitochondria, characterized by recurrent gene loss or functional relocation to the nuclear genome.

## 4 Discussion

At 2,422,778 bp, the mitochondrial genome of *L. williamsii* is exceptionally large and structurally fragmented, comprising 57 contigs that resolve into five linear molecules following decatenation. This architectural complexity aligns with the remarkable structural diversity of angiosperm mitochondrial genomes, which frequently deviate from the classical single circular model (Sloan et al., 2012; Wu et al., 2015; Szandar et al., 2022). The expanded genome size and fragmented organization likely reflect extensive repeat-mediated recombination, a hallmark of plant mitochondrial evolution that drives structural diversification and genome expansion (Gualberto and Newton, 2017). Relative to other members of Caryophyllales, the mitochondrial genome of *L. williamsii* is markedly enlarged with a length of 2,422,778 bp and a GC content of 42.84%. For instance, its size substantially exceeds that of recently published Cactaceae mitogenomes, including *Selenicereus monacanthus* (2,290,019 bp, GC content 43.37%) and *Pereskia aculeata* (515,187 bp, GC content 44.05%), as well as mitogenomes of other arid-adapted succulents such as *Sesuvium portulacastrum* (392,221bp, GC content 42.17%) and *Tetragonia tetragonoides* (347,227 bp, GC content 43.84%) (Li et al., 2020; Hu et al., 2021; Lu et al., 2023; Zhang et al., 2023). In terms of genomic composition, all these Cactaceae mitogenomes share a similar GC content (approximately 42–45%), yet *L. williamsii* harbors a considerably higher abundance of repetitive sequences and a more fragmented multi-chromosomal architecture compared with the simpler structures reported for *S. monacanthus* and *P. aculeata*. Among arid-adapted Caryophyllales, such expanded and repeat-rich mitogenomes appear to be a recurring feature, although the evolutionary forces driving these expansions may vary across lineages. This expansion is primarily driven by lineage-specific repeat accumulation and intracellular DNA transfer, a pattern consistent with evolutionary trends observed in other xerophytic and succulent plant lineages (Carpena et al., 2023).

We identified 587 simple sequence repeats, 124 tandem repeats, and 5,519 interspersed repeats ≥30 bp, dominated by forward and palindromic repeats. Such abundant repetitive elements are known to promote high-frequency intra- and intermolecular recombination, hereby driving the dynamic structural evolution characteristic of plant mitochondrial genomes (Gualberto and Newton, 2017). The prevalence of A/T-rich mononucleotide and tetranucleotide SSRs reflects the typical nucleotide composition bias of plant organellar genomes (Thiel et al., 2003; Wang et al., 2023). These repetitive loci, particularly the SSRs, represent informative molecular markers for future *L. williamsii* population genetic studies, germplasm characterization, and conservation management, as this species is increasingly imperiled by illegal harvesting and habitat degradation (Yesson et al., 2011; Hwang et al., 2025).

Codon usage analysis revealed a pronounced bias toward A/U-ending codons, a feature conserved across land plant mitochondrial genomes (Liu et al., 2025; Wang et al., 2025). ENC and neutrality plots demonstrated that codon usage preferences in *L. williamsii* are shaped predominantly by natural selection rather than mutational drift. This pattern mirrors findings in *I. longiauritus* and other angiosperms, where selective pressures related to translational efficiency and protein stability outweigh neutral mutational effects (Sueoka, 1988; Romero et al., 2000; Liu et al., 2025). The observed hierarchical distribution of GC content (GC1 > GC2 > GC3) further indicates conserved evolutionary constraints on codon positions that preserve amino acid hydrophobicity and protein stability properties critical for mitochondrial function under environmental stress (Zhang et al., 2024).

We detected 431 C-to-U RNA editing sites across 32 protein-coding genes, the majority of which increase the hydrophobicity of the encoded proteins. This strong bias toward C-to-U conversions is a nearly universal feature of land plant mitochondria, where RNA editing restores conserved amino acid residues and ensures functional protein synthesis (Mower, 2005; Small et al., 2023). Editing density was highest in *nad2* and absent in *cox1*, a gene-specific pattern documented in other plant lineages (Lu and Li, 2024; Chen et al., 2025) that suggests differential selective constraints on the RNA editing machinery. We propose that RNA editing contributes to the maintenance of mitochondrial activity under the extreme arid and high-temperature conditions typical of the Chihuahuan Desert (Ibarra-Laclette et al., 2015; Soto-Trejo et al., 2024).

Homology-based searches identified 18 chloroplast-derived DNA segments integrated into the *L. williamsii* mitochondrial genome, with a cumulative length of 31,614 bp and encompassing 19 full-length chloroplast genes. Intracellular gene transfer is a widespread evolutionary process in plants that contributes significantly to mitochondrial genome expansion and structural innovation (Keeling and Palmer, 2008; Bock, 2010). Most transferred regions encode photosynthesis-related genes and tRNAs, which are generally nonfunctional in the mitochondrial context but represent genomic relics of historical inter-organellar interactions. Comparable patterns of chloroplast-to-mitochondrion DNA transfer have been documented across angiosperms, including bamboos and figs, highlighting the dynamic evolutionary interplay between the two organellar genomes (Timmis et al., 2004; Straub et al., 2013; Liu et al., 2025; Wang et al., 2025).

Phylogenetic analysis based on 30 conserved mitochondrial genes robustly places *L. williamsii* as the sister species to *Mammillaria huitzilopochtli* within a strongly supported Cactaceae clade. This topology is congruent with traditional taxonomic classifications and with phylogenies derived from chloroplast data in the Caryophyllales (Yesson et al., 2011; Cauz-Santos et al., 2020). These results validate the utility of mitochondrial genes for resolving deep phylogenetic relationships in Cactaceae, a family in which morphological convergence and hybridization have long confounded systematics (Adrienne et al., 2016; Hwang et al., 2025). The close relationship between *Lophophora* and *Mammillaria* reinforces their placement within Cactoideae and illustrates the power of mitochondrial genomics to clarify evolutionary relationships in taxonomically challenging groups (Wang et al., 2024; Lin et al., 2025).

Selective-pressure analysis revealed that most core mitochondrial genes, including *atp1*, *cox1*, and *nad5*, are under strong purifying selection (Ka/Ks < 0.5), consistent with their essential roles in oxidative phosphorylation and mitochondrial homeostasis (Adams and Palmer, 2003; Bi et al., 2020). By contrast, *atp8* and *matR* exhibited Ka/Ks > 1, indicating positive selection. Genes under positive selection are frequently implicated in adaptive evolution, stress tolerance, or lineage-specific diversification (Fay and Wu, 2003; Zhao et al., 2022). Interestingly, *atp8* encodes a subunit of the mitochondrial ATP synthase complex, and its adaptive modification may influence the efficiency of ATP synthesis under abiotic stress conditions such as drought (Contreras-Díaz et al., 2023). Meanwhile, *matR* encodes a maturase involved in RNA splicing, and its positive selection could reflect optimization of mitochondrial gene expression in response to environmental challenges (Sultan et al., 2016; Mizrahi et al., 2022). Given that *L. williamsii* is endemic to the arid Chihuahuan Desert, it is plausible that the positive selection of these two genes reflects adaptive evolution to prolonged drought and high-temperature stress, although direct functional validation remains to be established (Cruz Plancarte and Solórzano, 2023). Pairwise sequence divergence analysis identified *atp9* as the most variable locus, while the majority of genes were highly conserved (Pi < 0.05), reflecting the characteristically slow evolutionary rate of plant mitochondrial coding sequences (Palmer and Herbon, 1988; Drouin et al., 2008).

Synteny analysis uncovered extensive homologous collinear blocks among the examined Cactaceae species, with the degree of collinearity generally decreasing with increasing phylogenetic distance. This pattern of diminishing shared synteny is consistent with observations in other plant lineages (Zhang et al., 2023; Liu et al., 2025). In addition to these shared homologous regions, substantial structural variations were also detected among the five species. Importantly, these observed structural differences should not be interpreted as evidence of recent interspecific recombination events. Rather, they most likely reflect the inherently high degree of structural plasticity that characterizes plant mitochondrial genomes, where extensive structural rearrangements and genomic variations are frequently observed even among different individuals within the same species, as demonstrated by recent pan-mitogenome studies (Soe et al., 2025; Mower et al., 2026). This dynamic nature is largely driven by repeat-mediated recombination (Cole et al., 2018; Qu et al., 2024). Comparative gene-loss analysis revealed sporadic losses of ribosomal protein genes and sdh4 across Cactaceae, a common trend in angiosperm mitochondria, where gene loss or functional transfer to the nucleus is frequent (Adams and Palmer, 2003; Ueda and Kadowaki, 2012). Detailed information on the pseudogenized genes identified in each species is provided in **Supplementary Table 1**. In contrast, genes involved in core energy metabolism remained universally conserved, emphasizing their indispensable biological functions (Manoj and Bazhin, 2021; Kwasniak-Owczarek and Janska, 2024).

Several limitations warrant consideration. Although long-read sequencing facilitated the assembly of the complex multi-branched mitochondrial genome structure, alternative isoforms and *in vivo* structural dynamics require experimental validation (Wick et al., 2015; Bi et al., 2024). Furthermore, the observed structural variations among species, while informative, must be interpreted with caution, as the highly dynamic nature of plant mitochondrial genomes limits the ability to infer specific evolutionary events from family-level comparisons alone. RNA editing sites were predicted computationally and require transcriptomic validation to confirm functional editing events (Mower, 2005; Small et al., 2023). Finally, expanded taxon sampling within Cactaceae will further enhance phylogenetic resolution and evolutionary inference (Yesson et al., 2011; Hwang et al., 2025). Additionally, broader taxon sampling within Cactaceae will be essential for further validating the observed correlation between synteny and phylogenetic distance.

In summary, the first complete mitochondrial genome of *L. williamsii* establishes a foundational genomic resource for dissecting the structural complexity, evolutionary dynamics, and adaptive evolution of mitochondrial genomes in Cactaceae. The extensive repetitive landscape, characteristic RNA editing profile, chloroplast DNA integration events, resolved phylogeny, and documented interspecific structural variations collectively delineate the distinctive evolutionary trajectory of this emblematic desert cactus and underscore the inherently dynamic nature of plant mitogenome architecture. These resources will facilitate future studies in conservation genetics, forensic identification, and evolutionary biology of *Lophophora* and related taxa, thereby contributing to the preservation of these ecologically and culturally significant plants, which face increasing anthropogenic threats (Ibarra-Laclette et al., 2015; Soto-Trejo et al., 2024).

## 5 Conclusion

Here we report the first fully assembled and annotated mitochondrial genome of *Lophophora williamsii*, addressing a longstanding gap in organellar genomic resources for Cactaceae. The mitogenome exhibits a highly complex multi-branched structure spanning 2,422,778 bp and contains abundant repetitive sequences. Codon usage bias is predominantly shaped by natural selection, and 431 C-to-U RNA editing sites were identified across 32 PCGs. We detected 18 chloroplast-derived DNA fragments transferred into the mitochondrial genome, providing evidence of extensive intracellular gene transfer. Phylogenetic analysis robustly places *L. williamsii* as the sister lineage to *Mammillaria huitzilopochtli* within Cactaceae. Most mitochondrial genes are subject to strong purifying selection, whereas *atp8* and *matR* show signatures of positive selection. Comparative synteny analysis further revealed extensive structural variation among Cactaceae mitochondrial genomes, consistent with the high degree of structural plasticity characteristic of plant mitogenomes. These findings advance understanding of mitogenome evolution in drought-adapted succulents and provide high-resolution molecular tools for species identification, conservation genetics, and forensic authentication of *Lophophora* and related taxa.

## Data Availability

The datasets presented in this study can be found in online repositories. The names of the repository/repositories and accession number(s) can be found in the article/**Supplementary Material**.
