# Supplementary material for: Assembly and comparative analysis of the first complete mitochondrial genome of Lophophora williamsii (Cactaceae)
Source: Front Plant Sci. 2026 Jun 2;17:1870314. doi: 10.3389/fpls.2026.1870314 (PMC13269275; doi:10.3389/fpls.2026.1870314)
Supplement: Supplementary file 1 [file Table1.docx]

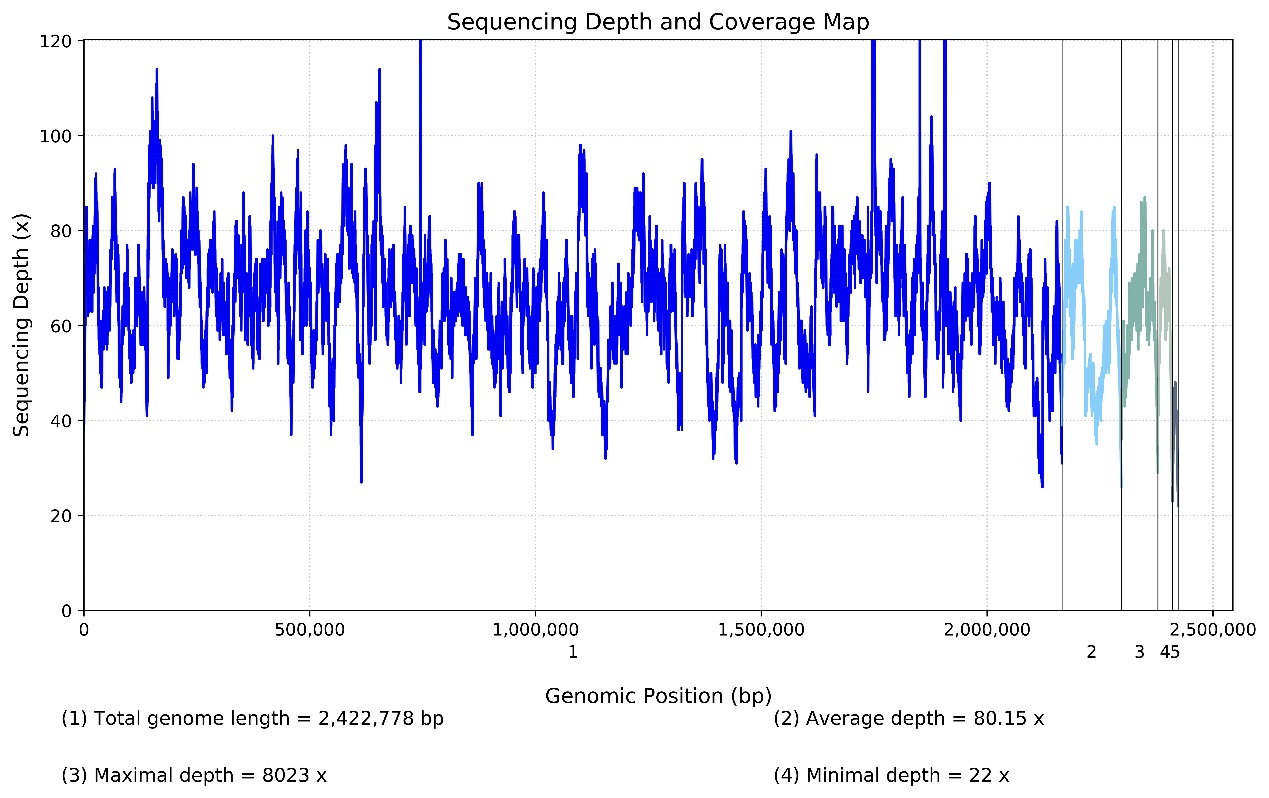


**Supplementary Figure S1.** Read mapping depth of the mitochondrial sequence. Clean read mapping depth is presented with blue bars. X and Y axis present nucleotide position of plastome and read mapping depth, respectively. The minimum and average mapping depth are 22× and 80.15×, respectively.
